# The association between sedentary behaviour and sarcopenia in older adults: a systematic review and meta-analysis

**DOI:** 10.1186/s12877-023-04489-7

**Published:** 2023-12-20

**Authors:** Yihan Mo, Yuxin Zhou, Helen Chan, Catherine Evans, Matthew Maddocks

**Affiliations:** 1 Cicely Saunders Institute of Palliative Care, Policy and Rehabilitation, Florence Nightingale Faculty of Nursing, Midwifery and Palliative Care, King’s College London, London, UK; 2 The Nethersole School of Nursing, Faculty of Medicine, The Chinese University of Hong Kong, Hong Kong, SAR China

**Keywords:** Sedentary behaviour, Sarcopenia, Systematic review, Meta-analysis

## Abstract

**Background:**

Sedentary behaviour is considered to contribute to sarcopenia when combined with physical inactivity. Whether sedentary behaviour is independently associated with sarcopenia remains controversial. The aim of this study is to explore the association between sedentary behaviour and sarcopenia in older adults in community and long-term care facility settings.

**Methods:**

Eight electronic databases including MEDLINE, PsycINFO, Wanfang were searched from inception until August 2023. The review included cross-sectional and longitudinal studies concerning the association between sedentary behaviour and sarcopenia among participants over 60 years old. Evidence was pooled by both random-effects meta-analysis and narrative synthesis. Subgroup analyses explored variation according to adjustment of physical activity, settings, and measurements of sedentary behaviour and sarcopenia. Quality assessment for individual studies was performed with the Joanna Briggs Institute (JBI) Critical Appraisal Checklist.

**Results:**

Seventeen articles (16 cross-sectional studies and 1 longitudinal study) of 25,788 participants from community or long-term care facility settings were included. The overall quality of the included studies was rated high. Meta-analysis of 14 cross-sectional studies showed that sedentary behaviour was independently positively associated with sarcopenia: pooled odd ratio 1.36 (95% confidence interval, 1.18–1.58). The independent positive association remained in subgroup analyses by adjustment of physical activity, settings, and measurements of sedentary behaviour and sarcopenia. The narrative analysis corroborated the findings of the meta-analysis and provided additional evidence suggesting that interruptions in sedentary periods were linked to a decreased likelihood of developing sarcopenia.

**Conclusions:**

The findings support the hypothesis that sedentary behaviour is independently positively associated with sarcopenia in older adults, providing vital indications for the development of strategies to prevent sarcopenia.

**Systematic review registration:**

The systematic review protocol has been registered with the PROSPERO database (CRD42022311399).

**Supplementary Information:**

The online version contains supplementary material available at 10.1186/s12877-023-04489-7.

## Background

Sarcopenia is defined as age-related loss of skeletal muscle mass plus loss of muscle strength and/or reduced physical performance [1]. The prevalence of sarcopenia worldwide is 10% among community-dwelling older adults while it is 38% among nursing home residents [2]. Sarcopenia is a strong predictor of a range of adverse clinical outcomes and is therefore an important public health concern. For example, it increases the risk of falls and fractures by approximately 90% in older people [3] and increase the risk of cognitive impairment two-fold [4]. It also increases the risk of disability, morbidity and mortality, and reduced quality of life for older adults [5–8]. Along with other risk factors, sedentary behaviour has been found to contribute significantly to sarcopenia when combined with physical inactivity [9, 10], and has been recommended to be an independent part from physical inactivity to achieve optimal musculoskeletal health [11, 12].

Sedentary behaviour is defined as any waking behaviour in a sitting, reclining or lying posture with low energy expenditure of ≤ 1.5 metabolic equivalent units (METs) [13, 14] while physical inactivity is when an individual does not perform a sufficient amount of physical activity to meet current age appropriate recommendations [11, 15]. Findings from systematic reviews suggest that greater sedentary time was related to an increased risk of all-cause mortality in older adults [16] and reduced cognitive function over the lifespan [17]. A meta-analysis provided support for the hypothesis that sedentary lifestyles are strong predictors of falls among older adults [18]. Some studies also indicated a relationship between sedentary behavior and metabolic syndrome, waist circumference, and overweightness/obesity [16]. An umbrella review reported that older adults (≥ 60 years) with physical inactivity are at an increased risk of all-cause and cardiovascular mortality, breast and prostate cancer, fractures, recurrent falls, disability in activities of daily life, functional limitation, cognitive decline, dementia, Alzheimer’s disease, and depression [19]. In addition, lower objectively measured sedentary behaviour and higher physical activity were reported to be associated with a better ability to complete activities of daily life and instrumental activities of daily life [20].

An important question is whether sedentary behaviour is independently associated with sarcopenia. Studies to date have reported inconsistent findings [21, 22], and the benefits of reducing sedentary time alone without increasing physical activity for people with sarcopenia remains unknown. This is largely because most exercise-based interventions only focus on increasing physical activity (e.g., resistance exercise training) with less consideration of reducing peoples’ sedentary behaviour [23–29]. The relationship between sedentary behaviour and sarcopenia has begun to be explored in recent studies. Some studies suggest that sarcopenia is an adverse outcome of sedentary behaviour [9, 30], whilst others suggest that sedentary behaviour is caused by sarcopenia and is a product of declining muscle mass and physical function [10, 31, 32].

Nevertheless, conflicting results are found across studies and positive association between sedentary behaviour and sarcopenia are not always found [22]. This paper is aimed to systematically examine the association between sedentary behaviour and sarcopenia among older adults.

## Materials and methods

### Protocol registration

We followed the principles of the Preferred Reporting Items for Systematic Reviews and Meta-Analyses 2020 statement (PRISMA 2020) [33] (Supplementary material 1) and registered the protocol with the PROSPERO database of systematic reviews (CRD42022311399).

### Search strategy and eligibility criteria

Eight bibliographic databases were searched, including five English language databases (MEDLINE via Ovid, Excerpta Medica (Embase) via Ovid, PsycINFO via Ovid, CINAHL via EBSCOhost, Web of Science) and three Chinese language databases (Chinese National Knowledge Infrastructure, Wanfang and SinoMed). Electronic searches were performed from their inception to 8th August 2023. The electronic search terms were designed to be broad and inclusive of sarcopenia and its components. The following text words were applied: “sarcopenia” “sedentary” “sitting”, as well as the Medical Subject Heading (MeSH) terms if applicable. Detailed search strategies for each database were presented in supplementary material 2. Search strategies were adapted for the eight different databases. There were no restrictions on publication date or language. We supplemented the electronic searches by checking the reference lists of included studies and by consulting experts to identify potentially eligible studies.

Eligibility criteria were (1) ***Study design***: Observational studies (cross-sectional studies and cohort studies) and baseline data of experimental studies (randomised control trials, quasi-randomised control trails, case-control studies); (2) ***Settings***: Community or long-term care facility setting; (3) ***Population***: Adults aged 60 years or older, without a neurological (e.g., motor neuron disease, stroke) or wasting condition (e.g., liver disease) affecting skeletal muscle health; (4) ***Sarcopenia***: Widely accepted diagnostic criteria for sarcopenia, including the European Working Group on Sarcopenia in Older Persons (EWGSOP) [9, 34], EWGSOP2 criteria [9], the Foundation for the National Institutes of Health (FNIH) criteria [35], the Asian Working Group for Sarcopenia (AWGS) criteria [36] or the strength, assistance in walking, rising from a chair, climbing stairs, and falls questionnaire (SARC-F) [37]; (5) ***Sedentary behaviour***: Objective or subjective measures of sedentary behaviour, recorded using any parameter, including sitting time, lying time, reclining time, counts per minute (CPM)-based intensity threshold values, and sedentary break times. Measurement tools could be self-reported questionnaires or any objectively physical devices (e.g., GT3X + accelerometers, activPAL device, etc.).

### Data management and selection process

Search results were imported into EndNote 20.2, duplicates were removed, and then imported into Covidence software (http://www.covidence.org) to screen and identify eligible publications. Two reviewers (YM, YZ) independently screened and reviewed a random sample of 20% of all titles and abstracts. At this stage of the process, regular reviewer meetings were held to compare decisions on eligibility, discuss any uncertainties, and reach consensus. At the end of this process, the two reviewers reached agreement on all the 20% of the studies. Finally, one reviewer (YM) screened the remaining 80% of the titles and abstract independently. Records that appeared to meet the criteria or with any uncertainty were further screened in full text. Full text records were reviewed by both reviewers (YM, YZ) independently and discussed when there was any disagreement on eligibility. A third reviewer (CE or MM or HC) was invited when the disagreement was unresolved.

### Data extraction and outcomes of interest

Data from included articles were extracted by two reviewers independently (YM, YZ) with the guidance of a data extraction template designed for the study. This form included the following information: (1) Article title, authors, year of publication, country; (2) Study design; (3) Participant description, including age, sex, ethnicity and medical conditions; (4) Setting, such as community or long-term care facility; (5) Sedentary behaviour definition and measurement method; (6) Sarcopenia definition and measurement method; (7) Main results – outcome of sarcopenia and explanatory variable of sedentary behaviour, and potential confounders such as age, sex, physical activity, nutritional status, and chronic diseases. When a study provided several adjusted models, the fully adjusted model was extracted; (8) Brief conclusion and limitations of the study. Corresponding authors of the articles were contacted in cases of missing information or data. The primary outcomes were the adjusted associations between sedentary behaviour and sarcopenia, expressed as Odd Ratio (OR) value or Relative Risk (RR) value or Hazard Risk (HR) value and 95% confidence intervals (CI). The secondary outcomes were (1) the relationship between sedentary behaviour and sarcopenic obesity, and/or components of sarcopenia, (2) sedentary break times and sarcopenia (sarcopenic obesity), and (3) sub-group analysis by adjustment of physical activity, setting, measure of sedentary behaviour, and measure of sarcopenia.

### Quality assessment of included studies

Two reviewers (YM, YZ) independently conducted the quality assessment of included studies using assessment tools accordingly. For cross-sectional studies, the Joanna Briggs Institute (JBI) Critical Appraisal Checklist for Analytical Cross-Sectional Studies (8 items) was used [38]. For cohort studies, the JBI Critical Appraisal Checklist for Cohort Studies (11 items) was used [38]. Each item was scored as 0 or 1 or 2 points; 0 = the aspect does not meet the requirements (No), 1 = the aspect has been mentioned but without a detailed description (Uncertain), 2 = the aspect has been described in detail comprehensively (Yes). The summary score, obtained by dividing the total score by the maximum possible score, was used to classify studies as high quality ≥ 70%, medium quality 40–69%, or low quality < 40%. Both reviewers (YM, YZ) recorded the score process and classification. When there was any unresolved disagreement on the quality of studies between the two reviewers after discussion, a third reviewer (CE or MM or HC) was invited to discuss to reach an agreement.

### Data synthesis

Data were synthesised using meta-analysis when studies presented (1) sedentary behaviour/time and dichotomous classifications of sarcopenia, and (2) were sufficiently homogenous from a clinical (i.e., population, outcome) and methodological (i.e., study design) point of view. The random-effect model was used to determine the pooled OR value for the association between sedentary behaviour and sarcopenia. Subgroup analysis was performed based on (1) adjustment of physical inactivity, (2) study setting, (3) measures of sedentary behaviour, (4) measures of skeletal muscle mass, muscle function, and (5) diagnostic criteria of sarcopenia. Narrative analysis was performed for data that did not meet the criteria for meta-analysis.

The statistical heterogeneity of the included studies was examined by the chi square-based Cochran’s Q statistic test and *I*^2^ statistic [39, 40]. *p* < 0.1 or *I*^2^ > 50% indicates significant heterogeneity [38]. Sensitivity analyses were used to test the effect of including different studies. For studies which were judged to be too clinically or methodologically heterogeneous, a narrative approach was used to synthesis the data [41]. To assess publication bias, funnel plots were inspected visually, and the Egger’s test was used. Statistical analyses were performed using the metan and metainf packages in the STATA v 15 (Stata Corp, College Station, TX). Two-tailed *p* values < 0.05 were considered statistically significant.

## Results

### Retrieval

**Fig. 1:**
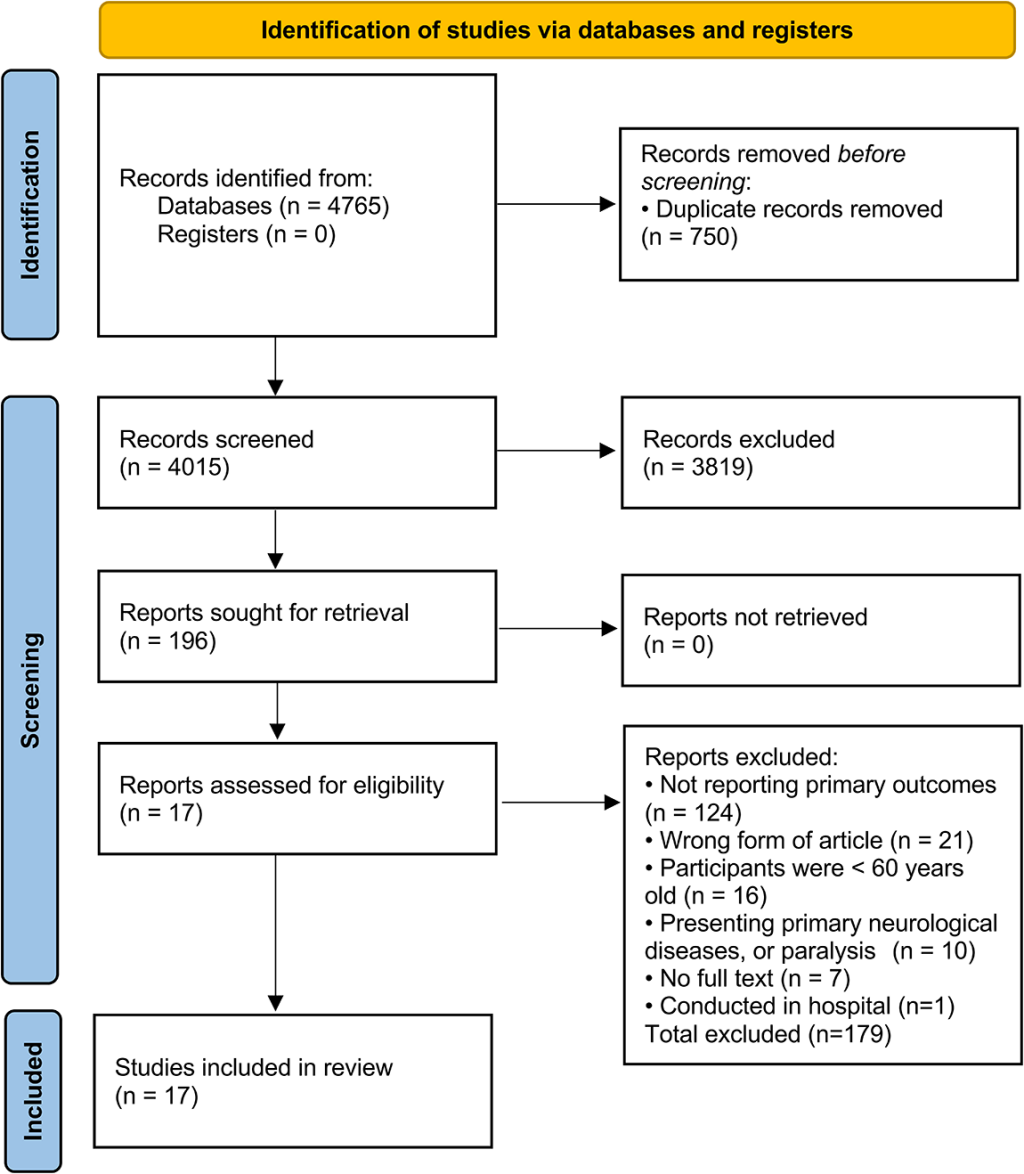
Flowchart for study selection process

A total of 4765 articles were identified through an initial literature search. After removing duplicates and screening of titles and abstracts, 196 full-text articles were assessed for eligibility. The main reason for ineligibility was the study not reporting the association between sedentary behaviour and sarcopenia. Finally, 17 articles [8, 10, 21, 22, 30–32, 42–51] were included (Fig. 1).

### Characteristics of included studies

Sixteen cross-sectional studies [8, 10, 21, 22, 30–32, 43–51] and one longitudinal study [42] were included. The 17 included articles comprised 25,788 participants with the age ranging from 60.0 to 92.7 years. The proportion of male and female participants in the total included sample were 45.6% and 54.4% respectively. In terms of the targeted population, 14 studies included community populations [8, 10, 21, 22, 32, 42–48, 50, 51], and three studies [30, 31, 49] recruited residents living in long-term care facilities. Regarding sarcopenia diagnostic criteria, nine studies [10, 21, 30–32, 42, 43, 45, 48] used the EWGSOP criteria [34], three studies [22, 44, 49] used EWGSOP2 criteria [9], and the remaining four articles diagnosed sarcopenia separately using the SARC-F questionnaire (n = 2) [47, 50], the AWGS criteria (n = 2) [46, 51] or FNIH criteria (n = 1) [8]. For the measurement of skeletal muscle mass, dual-energy X-ray absorptiometry (DXA) (n = 5) [8, 22, 32, 43, 48], bioelectrical impedance analysis (BIA) (n = 6) [30, 31, 44, 46, 49, 51], and anthropometrics and equations (n = 4) [10, 21, 42, 45] were used. Another two studies [47, 50] used SARC-F questionnaire to diagnose sarcopenia without a direct measurement of skeletal muscle mass. For measurement of skeletal muscle strength, hand grip strength (HGS) were used in most of the included studies except one study using leg muscle strength [32] and two studies not measuring this aspect [47, 50]. The assessment tools for sedentary behaviour were mainly self-reported questionnaires (n = 12) [10, 30–32, 42, 43, 45–48, 50, 51], such as the International Physical Activity Questionnaire [52] (IPAQ) [30, 31, 43, 45, 47, 48], and five studies adopted objective measurement of physical activity by using ActiGraph accelerometer [8, 21, 22, 44, 49]. All studies were classified as high-quality with scores ranging from 81.25 to 100%. Detailed study characteristics and the quality assessment results are shown in Tables 1 and 2 respectively.

### Meta-analysis results

Fourteen cross-sectional studies [8, 10, 21, 30–32, 43–47, 49–51] with a total of 21,989 participants were pooled in the meta-analysis. In five studies [10, 46, 47, 50, 51] that grouped participants according to sedentary time in several categories, OR values from the group with the longest sedentary time were used. After adjusting for confounders including sex, age, BMI, education, physical activity, chronic diseases, etc., the pooled adjusted OR value of the association between sedentary behaviour and sarcopenia extracting from 14 articles [8, 10, 21, 30–32, 43–47, 49–51] was 1.36 (95%CI, 1.18–1.58). There was significant heterogeneity (*p* < 0.001, *I*^*2*^ = 80.2%) across studies. The high heterogeneity may result from with or without adjustment of physical activity, different settings, various measurements of sedentary behaviour and sarcopenia, different diagnostic criteria of sarcopenia. We further conducted subgroup analyses based on these factors.

**Fig. 2:**
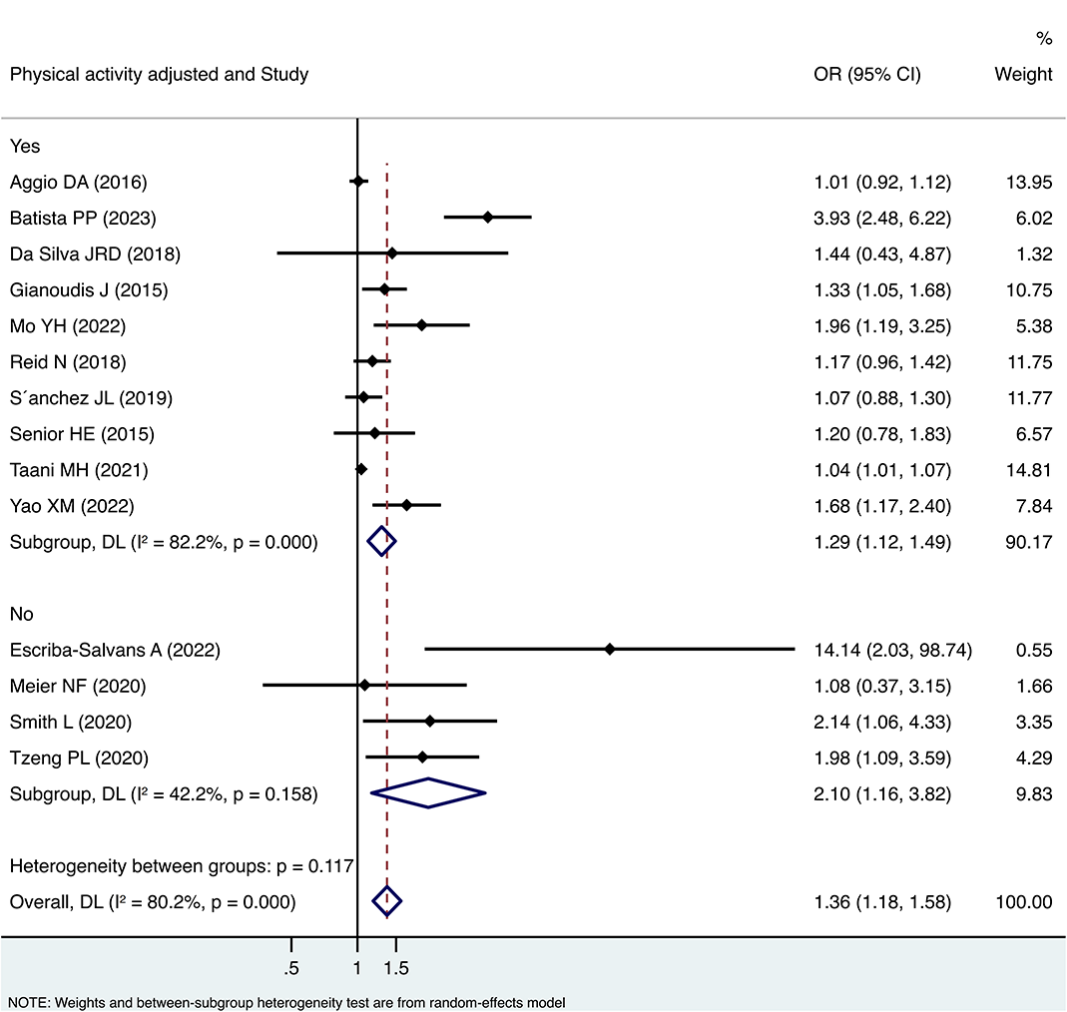
Forest plot of the associations between sedentary behaviour and sarcopenia by subgroup analysis based on with or without adjustment for physical activity

**Fig. 3:**
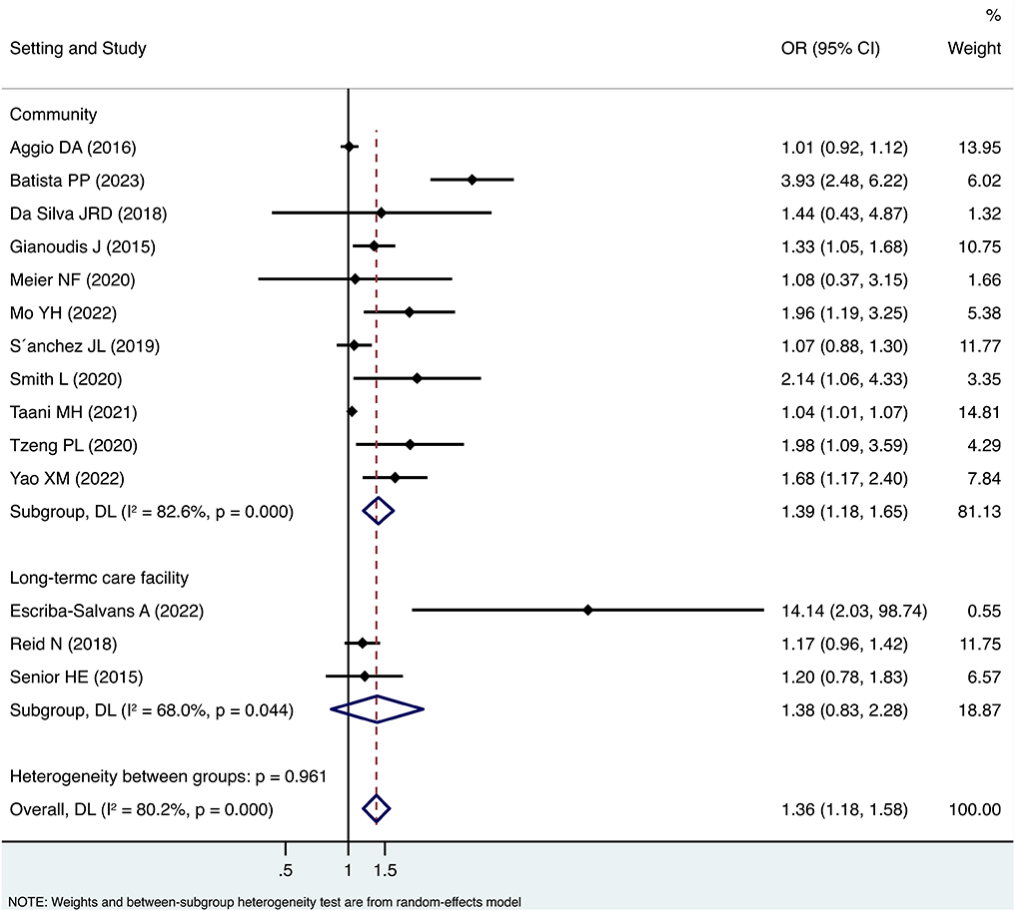
Forest plot of the associations between sedentary behaviour and sarcopenia by subgroup analysis based on settings

**Fig. 4:**
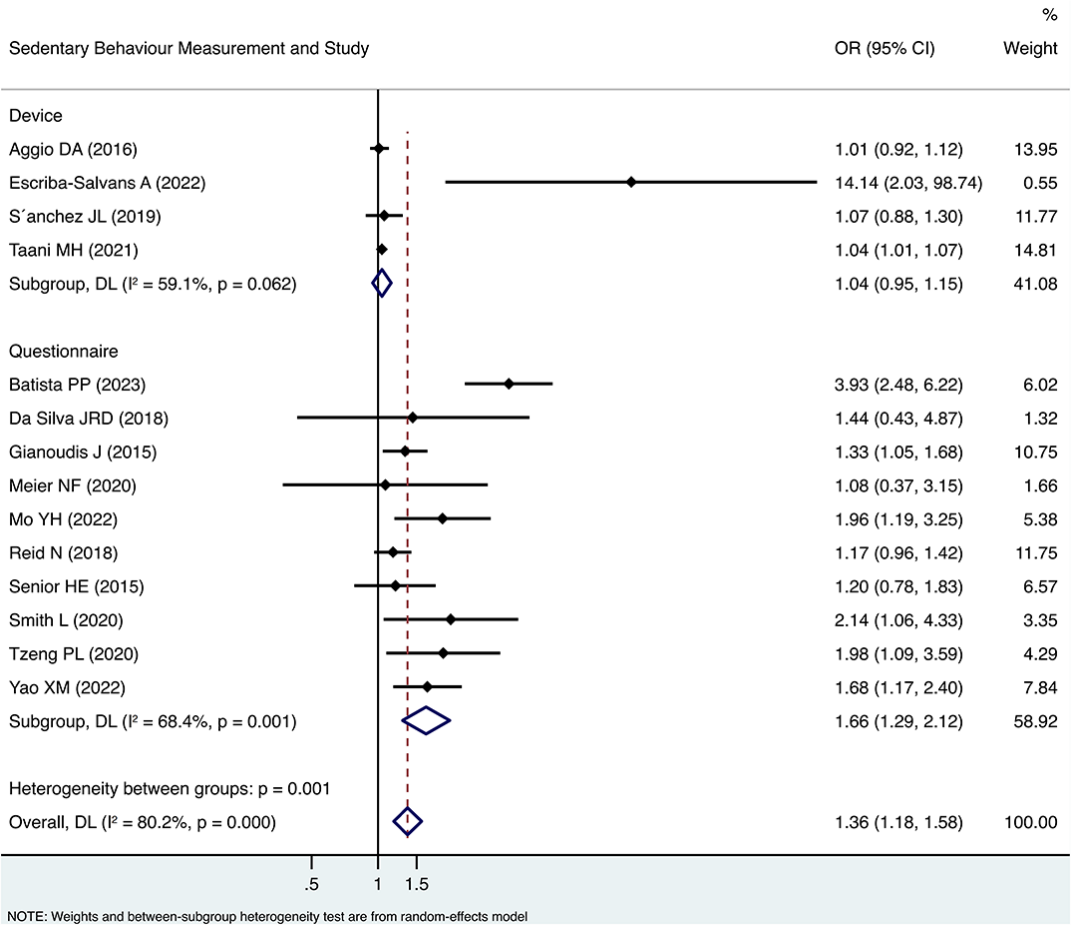
Forest plot of the associations between sedentary behaviour and sarcopenia by subgroup analysis based on sedentary behaviour measurement

Subgroup analyses showed a stronger association between sedentary behaviour and sarcopenia without adjustment for physical activity (OR 2.10, 95%CI 1.16, 3.82) than with adjustment for physical activity (OR 1.29, 95%CI 1.12, 1.49). However, the difference was not statistically significant ((between groups *p* = 0.117) (Fig. 2). As to different setting, studies enrolling community-dwelling older adults (OR 1.39, 95%CI 1.18, 1.65) tended to find a similar association between sedentary behaviour and sarcopenia with studies enrolling residents in long-term care facility (OR 1.38, 95%CI 0.83, 2.28) (between groups *p* = 0.961) (Fig. 3). In terms of measurement of sedentary behaviour, studies using self-reported questionnaire showed higher OR value (OR 1.66, 95%CI 1.29, 2.12) than the studies using objective physical measurement (OR 1.04, 95%CI 0.95, 1.15). The difference was statistically significant (between groups *p* = 0.001) (Fig. 4).

Additional subgroup analysis for different muscle mass and physical performance measurements also supported the positive association between sedentary behaviour and sarcopenia. The group not measuring skeletal muscle mass nor physical performance (the two studies using SARC-F questionnaire defining sarcopenia) demonstrated a significant stronger association (OR 2.87, 95%CI 1.47, 5.60). In contrast, the group that measured muscle mass using DXA (OR 1.17, 95%CI 1.00, 1.35) and the group that measured physical function using SPPB (OR 1.18, 95%CI 0.98, 1.40) showed the lowest OR values in the corresponding subgroup analysis (Table 3). As to different sarcopenia diagnostic criteria, we only conducted a subgroup meta-analysis for EWGSOP criteria because only it was used in more than three studies within the 14 studies included in meta-analysis part of this study, with a pooled OR of 1.17 (95%CI 1.01, 1.34).

The association between sedentary behaviour and sarcopenia was further confirmed by sensitivity analysis. Sensitivity analysis was performed repeatedly by removing one study each time, with the pooled OR fluctuating between 1.22 (95%CI 1.08–1.36) and 1.51 (95%CI 1.22–1.85). Removing any single study did not change the overall meta-analysis results indicating the finding as robust and reliable. The asymmetric funnel plot indicated a possible publication bias (Supplementary material 3), supported by the Egger’s test (*p* = 0.002).

### Narrative synthesis results

Three studies [22, 42, 48] were only synthesised narratively due to heterogeneity. Overall, these studies supported a positive association between sedentary behaviour and sarcopenia, and sarcopenic obesity. In a 24-month prospective study, older adults who presented slow gait speed during the follow-up and with sedentary behaviour presented a higher risk of sarcopenia, independent of physical activity levels, age, and sex (HR 1.30, 95% CI 0.40, 4.24). At the same time, older adults who remained sedentary at 24-month follow-up also independently demonstrated a higher risk of sarcopenic obesity [42]. The study enrolling Quilombola, Afro-Brazilian residents, as participants found that older adults who were irregularly active or sedentary (not discriminated) were at least six times more likely to develop sarcopenia than those who were assessed to be active to very active [48]. Only one study examining the relationship between accelerometer-determined sedentary behaviour and probable or confirmed sarcopenia (not discriminated) in community-dwelling older adults demonstrated no association after multivariable adjustment [22].

Additional data from three studies included in the meta-analysis [8, 21, 32] also contributed to the narrative analysis, supporting the negative association between breaks in sedentary time and risk of sarcopenia, and sarcopenic obesity. Using an isotemporal substitution model, one study [8] found that the reallocation of one hour per day of sedentary behaviour with moderate-to-vigorous physical activity (MVPA) lowered sarcopenia risk (OR 0.52, 95%CI 0.36–0.75; P < 0.001), and when moderate-to-vigorous physical activity was substituted with sedentary behaviour, the sarcopenia risk was raised (OR 1.92, 95%CI 1.33, 2.77; P < 0.001). A cross-sectional study in a community setting suggested that self-reported breaks in sedentary time were associated with a lower risk of sarcopenia (OR 0.26, 95%CI 0.05, 1.39) [32]. Another cross-sectional study also found that for community-dwelling older men, sedentary breaks were marginally associated with a reduced risk of sarcopenic obesity (RR 0.84 [95% CI 0.71, 0.99]). In addition, it also reported a marginal association between sedentary time and increased risk of severe sarcopenia (RR 1.07 [95% CI 0.91, 1.26]) and sarcopenic obesity (RR 1.18 [95% CI 0.99, 1.40]), independent of physical activity levels [21].

## Discussion

This systematic review and meta-analysis highlighted the independent positive association between sedentary behaviour and sarcopenia, regardless of adjustment of physical activity, community or long-term care facility settings, or different measurements of sedentary behaviour and sarcopenia. Our findings align with recent studies. For instance, a systematic review demonstrated that sedentary behaviour and physical inactivity is strongly associated with reduced skeletal muscle strength and diminished muscle power, which are critical characteristic of sarcopenia [53]. A 2-year longitudinal cohort study also suggested that older adults who maintained sedentary behaviour and exhibited low gait speed during the follow-up were at a greater risk of sarcopenia [42].

Lower gait speed of older adults with sarcopenia may be a possible factor which explains the association between sedentary behaviour and sarcopenia. It has been found that low gait speed is associated with high sedentary behaviour [54]. On the one hand, older adults with a slower gait speed are more likely to have poorer functional status and overall health [55], and to experience multiple falls [56], therefore, they are prone to choose sedentary behaviour in their daily life. On the other hand, accumulating sedentary time for prolonged time is independently related to the disuse of muscle and tendon [57, 58]. Severe muscle disuse induces rapid muscle atrophy [59, 60], leading to a vicious circle. Several plausible physiological mechanisms could also offer insight into the association. First, high level of sedentary behaviour may result in diminished muscle protein synthetic response by reducing muscle anabolic sensitivity [11]. The decreased sensitivity of muscles to anabolic signals is likely a significant factor in the muscle loss and decline in physical function (i.e., sarcopenia) [11]. Second, prolonged sitting time could enhance the levels of chronic low-grade inflammation [61] and increase deep adipose tissue and visceral adiposity [29], which have been shown to promote muscle wasting, ultimately stimulating protein catabolism and suppressing muscle synthesis [62].

The subgroup analysis demonstrated the adjustment of physical activity do not result in statistically significant difference on the independent association between sedentary behaviour and sarcopenia. Former studies also suggested that prolonged involvement in sedentary activities detrimentally affects skeletal muscle mass and functional abilities among older adults, regardless of their engagement in physical activity [31, 32]. Hence, it is recommended to consider sedentary behaviour and physical inactivity as two distinct risk factors, each requiring targeted interventions to attain optimal musculoskeletal health [11, 63, 64]. Results of narrative analysis found that breaks in sedentary time and replacing sedentary time with physical activity contribute to reduced risk of sarcopenia [8, 21, 32]. This is supported by the study that found breaking-up sedentary time is associated with physical function in older adults [65]. Further research also indicates that breaking up sedentary time regularly with a sufficient level of movement that goes beyond a simple muscular contraction (such as walking instead of merely standing) could potentially be effective in maintaining skeletal muscle anabolic sensitivity, muscle mass, and physical function in older adults [66].

The findings from subgroup analysis indicated that sedentary behavior raised the risk of sarcopenia by around 40% in both community-dwelling older adults and long-term care facility residents. Studies have indicated that individuals over the age of 60 spend around 80% of their waking hours engaged in sedentary activities, equating to approximately 8 to 12 h each day [67–69]. Particularly among residents in long-term care facilities, a significant 85% of their waking hours are occupied by sedentary activities [70]. The prevalence of sarcopenia among long-term care facility residents (38%) is also higher than in the community-dwelling older adults (10%) [2]. Given the functional limitations and multimorbidity of the majority of residents in long-term care facilities, interventions that target the reduction of sedentary behavior rather than demanding physical exercise might be a more significant, practical and approachable approach to combat sarcopenia. This is also well reflected in recent recommendations and guidelines which make it a priority to reduce sedentary behaviour among all long-term care facility residents [63, 64]. Notably, we only identified three cross-sectional studies conducted in long-term care facilities, more studies with diverse study design are warranted to explore sedentary behaviour and sarcopenia in long-term care facilities.

Results of subgroup analysis showed that sedentary behaviour significantly increased the risk of sarcopenia by 66% in the group using self-reported questionnaires, which is almost sixteen-fold higher than that of the group using objective physical devices measurement (4%). The difference may be attributed to recall bias and a low correlation between subjective and objective measures of sedentary time [71, 72]. Some included studies using self-reported questionnaires to measure sedentary time only used a single question “how much time did you usually spend on sitting during the last 7 days” [30, 31, 47, 73]. However, this broad question without detailed prompts could be difficult for older adults to recall [74], and tends to misestimate their sedentary time compared to objective measures [75, 76]. To increase the validity of self-reported questionnaires, additional detail of types or examples of activities on a daily basis and a visual analogue scale are recommended [71, 76]. Ecological momentary assessment (EMA) gathering real-time self-reports of behaviours, contexts, emotional states, and perceptions in naturalistic setting may also be an effective way to reduce recall bias [77]. On the other hand, the potential motivational effect of sedentary behaviour measurement devices may diminish the association between sedentary behaviour and sarcopenia. Wearing a device that monitors activity time are used to enhance intervention effect and compliance [78, 79]. The feeling of novelty and being supervised with a physical device may motivate older adults to increase their activity, termed reactivity [80]. From this perspective, objective measurement instruments of sedentary behaviour can also serve as a part of sedentary behaviour intervention.

### Strengths and limitations

There are some strengths of this review. First, the strict inclusion of studies which used validated sarcopenia definitions, taking confounders into consideration enhanced the rigour of our results. Besides, searching across three widely used Chinese bibliographic databases provided greater coverage of possible related studies. Several limitations of our review should be addressed. First, only one longitudinal study meeting our eligibility criteria was included. Due to the heterogeneity, only cross-sectional studies were included in the meta-analysis. More longitudinal studies focusing on this topic are warranted. Second, almost all the included studies only reported the OR/RR/HR value between sedentary behaviour presented in terms of categorical format and sarcopenia, rather than continuous sedentary time. This may have caused some bias of different cut-off points when synthesising. Furthermore, most studies included in the meta-analysis were adjusted for age, chronic diseases and physical activity when exploring the association between sedentary behaviour and sarcopenia, but nutritional status, which is a key risk factor for sarcopenia [9, 81], was not commonly adjusted for. A standard set of confounders covering the main risk factors of sarcopenia is recommended in further studies. Finally, even though studies which comprised populations from both the community and long-term care facility settings were included, only three studies from long-term care facilities were identified. This limits the generalizability of the findings across settings and reveals the priority for future research in long-term care facilities.

## Conclusion

In conclusion, sedentary behaviour is independently positively associated with sarcopenia in older adults, regardless of adjustment of physical activity, settings, measurements of sedentary behaviour and sarcopenia. The findings provide vital indications for the development of strategies to prevent sarcopenia.

**Table 1:** Characteristics of the included studies

Study	Study design	Participant’s description	Setting	Prevalence of sarcopenia	Diagnostic criteria of sarcopenia	Measures of sarcopenia	Prevalence of sedentary behaviour or sedentary time	Measures of sedentary behaviour	Confounders
Aggio DA, 2016, Britain (21)	Cross-sectional study	*Sample size*: 1286 *Age, mean (SD)*: 70–92 years old *Male, n (%)*: 1286 (100%) *Ethnicity*: 100% white *Medical conditions*: not stated	Community (from primary care practice)	Sarcopenia: 14.2% Severe sarcopenia: 5.4%	EWGSOP	LMM (Equation) + LMS (HGS) or LPP (3 m-GS)	Mean (min/day): Non-sarcopenia: 610.9 Sarcopenia: 614.1	Actigraph, GT3X accelerometer over the hip for 7 days	Age, wear time, season, region, social class, number of chronic conditions, smoking, alcohol, height, waist circumference
Batista PP, 2023, Brazil (50)	Cross-sectional study	*Sample size*: 1482 *Age*: 68.17 ± 11.17 Male, n (%): 385 (26%) *Ethnicity*: not stated *Medical condition*: 56.3% with *≥ 2 Comorbidities*	Community (at home)	17.1%	SARC-F questionnaire	None	Sitting time, % < 4 h: 28.8 5–7 h: 31.1 8-10 our: 16.9 > 10 h: 23.2	One question about the duration of sitting activities in the prior week	Age, sex, marital status, education, income, occupation
Da Silva JRD, 2018, Brazil (45)	Cross-sectional study	*Sample size*: 101 *Age*: ≥60 years old *Male, n (%)*: 38 (41.8%) *Ethnicity*: not stated *Medical conditions*: not stated	Community (at home)	23.1%	EWGSOP	LMM (Equation) + LMS (HGS) or LPP (TUG)	Sitting time ≥ 7 h/day: 73.6%	Questions from IPAQ	Diabetes mellitus, smoking, physical activity, daily caloric intake, daily protein intake, serum vitamin D concentration, and ACE I/D gene polymorphism
Escriba-Salvans A 2022, Spain (49)	Cross-sectional study	*Sample size*: 104 *Age*: 84.6 ± 7.8; *Male, n (%)*: 19(18.3%) *Ethnicity*: not stated *Medical conditions*: 32.5% with hypertension, 32.5% with dementia, 22.5% with cardiac pathology, 16.8% with depression, 16.8% with diabetes, etc.	Long-term care facility	18.3%	EWGSOP2	SARC-F + LMS (HGS) + LMM (BIA)	Sedentary behaviour time > 85% in total time: 31.6%	activPAL3TM activity monitor (PAL Technologies Ltd., Glasgow, UK) at mid-thigh	Age, sex, nursing home type
Gianoudis J, 2015, Australia (32)	Cross-sectional study	*Sample size*: 162 *Age*: 67.5 ± 6.0 *Male, n (%)*: 43 (26%) *Ethnicity*: not stated *Medical conditions*: 70% with at least one kind chronic disease, at risk for falls and fracture	Community (at home)	16%	EWGSOP	LMM (DXA) + LMS (Leg muscle strength) or LPP (TUG)	TV viewing time, h/day: 2.7 ± 1.6 Total sitting time, h/day: 6.1 ± 2.1	7-day recall questionnaire	Age, sex, physical activity, chronic disease, medications, smoking, sedentary job, total BFM
Meier NF, 2020, United States (43)	Cross-sectional study	*Sample size*: 304 *Age*: 72.2 ± 5.8 *Male, n (%)*: 182 (40%) *Ethnicity*: 100% Caucasian *Medical conditions*: not stated	Community (at home)	10.9%	EWGSOP	LMM (DXA) + LMS (HGS) or LPP (GS)	Mean (SD) (h/day): Not sarcopenic: 10.6 (4.0) Sarcopeniac: 12.0 (5.1)	Questions from IPAQ	Age, sex, BMI, education, income, smoking, drinking, chronic conditions, depressive symptoms
Mo YH, 2022, China (46)	Cross-sectional study	*Sample size*: 1050 *Age*: 70.3 ± 7.5 *Male, n (%)*: 347 (33.0%) *Ethnicity*: Yellow (100%) *Medical conditions*: 15.3% with diabetes, 43.4% with hypertension	Community (from Community Elderly Activity Centre)	25.0%	AWGS	LMM (BIA) + LMS (HGS) or LPP (GS)	Sitting time without breaks classification < 2 h: 23.1% 2–4 h: 27.7% > 4 h: 49.2%	Questions from PASE	Age, BMI, marital status, dietary diversity, physical activity
Reid N, 2018, Australia (31)	Cross-sectional study	*Sample size*: 102 *Age*: 84.5 ± 8.2 *Male, n (%)*: 31 (30.4%) *Ethnicity*: not stated *Medical conditions*: Low care, high care, or people with dementia residing in a secure dementia unit	Long-term care facility	40.2%	EWGSOP	LMM (BIA) + LMS (HGS) or LPP (SPPB)	Total sitting time(h/day) Mean (SD): 12.9 (3.0)	Question from IPAQ	Age and sex, physical activity, nutritional status and BMI
Ribeiro SV, 2020, Brazil (42)	Cohort study	*Sample size*: 395 *Age*: ≥60 *Male, n (%)*: 59 (28.0%) *Ethnicity*: White: 64.0%, Brown/black: 24.2%, Asian: 11.8% *Medical conditions*: not stated	Community (at home)	8.5%	EWGSOP	LMM (Equation) + LMS (HGS) + LPP (GS)	Not stated	Self-report questionnaire	Age and sex
Sánchez JL, 2019, Spain (8)	Cross-sectional study	*Sample size*: 497 *Age*: 78.08 ± 5.71 *Male, n (%)*: 219 (45.7%) *Ethnicity*: not stated *Medical conditions*: 65.4% with hypertention, 22.9% with Type 2 diabetes mellitus, 54.3% with dependency for IADL (Lawton index)	Community (further detail not stated)	23.3%	FNIH	LMM (DXA) + LMS (HGS) + LPP (GS)	Total sitting time (h/day) Mean (SD): Non sarcopenia 6.82 (1.57); Sarcopenia 7.53 (1.63)	Actigraph, GT3X accelerometer for 7 days	LPA and MVPA
Scott D, 2021, Swedish (22)	Cross-sectional study	*Sample size*: 3334 *Age*: 70.1 ± 0.1 *Male, n (%)*: 1647 (49.4%) *Ethnicity*: not stated *Medical conditions*: some participants with stroke	Community (at home)	1.8%	EWGSOP 2 -probable and confirmed sarcopenia	Probable: LMS (HGS) Confirmed: LMM (DXA) + LMS (HGS) + LPP (TUG)	Total sitting time (h/7 days) Mean (SD): Non sarcopenia 58.7 (12.8); Sarcopenia 59.5 (15.3)	Actigraph, GT3X accelerometer for 7 days	Gender, BMI, smoking status, time spent on LPA and MVPA
Senior HE, 2015, Australia (30)	Cross-sectional study	*Sample size*: 102 *Age*: 84.5 ± 8.2 *Male, n (%)*: 31 (30.7%) *Ethnicity*: not stated *Medical conditions*: Low care, high care, or people with dementia residing in a secure dementia unit	Long-term care facility	40.2%	EWGSOP	LMM (BIA) + LMS (HGS) or LPP (SPPB)	Total sitting time (h/day) Mean (SD): 12.9 (3.0)	Questions from IPAQ	BMI, SPPB, nutritional status
Sinesio Silva Neto L, 2016, Brazil (48)	Cross-sectional study	*Sample size*: 70 *Age*: 65.58 ± 6.67 *Male, n (%)*: 31(44.3%) *Ethnicity*: 100% quilombola *Medical conditions*: not stated	Community (at home)	10.0%	EWGSOP	LMM (DXA) + LMS (HGS) or LPP (GS)	Infrequently active + sedentary: 12.9%	Score of IPAQ-SV	Sex, age, education, cognitive function, weight, hight, BMI, WC, WHR, FM%, ASMM, HGS, GS
Smith L, 2020, Low and Middle-Income Countries (10)	Cross-sectional study	*Sample size*: 14,585 *Age*:72.6 ± 11.5 *Male, n (%)*: 6563(45%) *Ethnicity*: Yellow: 7801, White and Hispanic: 3325, Black: 3459 *Medical conditions*: not stated	Community (at home)	15.7%	EWGSOP	LMM (BIA) + LMS (HGS) or LPP (4 m-GS)	Sitting time classification (h): 0-<4: 43.3% 4-<8: 40.8% 8-<11: 11.8% ≥ 11: 4.1%	Single question from GPAQ	Age, sex, wealth, education, smoke, drink, chronic disease, BMI, physical activity
Taani MH, 2021, United States (44)	Cross-sectional study	*Sample size*: 96 *Age*: 82.5 ± 7.4 *Male, n (%)*: 17 (17.7%) *Ethnicity*: 81.3% White or Caucasian, 16.7% Black or African American, 2.1% American Indian/Alaska Native *Medical condition*: not stated	Community (Continuing care retirement communities)	37.5%	EWGSOP2	LMM (BIA) + LMS (HGS) or LPP (GS)	Total sitting time for 7 days (h): 519.1 ± 77.8	Actigraph, GT3X accelerometer for 7 days	Protein, Caloric intake, LPA, MVPA, self-efficacy and expectations regarding aging
Tzeng PL, 2020, Taiwan (47)	Cross-sectional study	*Sample size*: 1068 *Age*: 65 to 92 years old *Male, n (%)*: 505 (47.3%) *Ethnicity*: not stated *Medical conditions*: not stated	Community (further detail not stated)	7.3%	SARC-F questionnaire	None	High (sitting ≥ 7 h/day): 167 (15.6%) Low (< 7 h/day): 901 (84.4%)	Single question from IPAQ-SV	Sex, age, residential geographic areas, education, marital status, employment, living status, BMI
Yao XM, 2022, China (51)	Cross-sectional study	*Sample size*: 1050 *Age*: ≥ 60 *Male, n (%)*: 347 (33%) *Ethnicity*: not stated Medical conditions: 43.4% with hypertension, 15.3% with diabetes, 14.6% with coronary heart disease	Community (from Community Elderly Activity Centre)	25.0%	AWGS	LMM (BIA) + LMS (HGS) or LPP (GS)	Sitting time without breaks classification < 2 h: 268 2–4 h: 300 > 4 h: 482	Questions from PASE	Age, Education, BMI, Calf circumference

SD, standard deviation; IADL, Instrumental Activities of Daily Living; EWGSOP, European Working Group on Sarcopenia in Older People; SARC-F, Strength, Assistance in walking, Rise from a chair, Climb stairs, and Falls; AWGS, Asian Working Group for Sarcopenia; FNIH, The Foundation for the National Institutes of Health; LMM, low muscle mass; LMS, low muscle strength; HGS, hand grip strength; LPP, low physical performance; GS, gait speed; TUG, time up to go; BIA, bioelectrical impedance analysis; DXA, dual-energy X-ray absorptiometry; SPPB, short physical performance battery; IPAQ, international physical activity questionnaire; PASE, physical activity scale for the elderly; IPAQ-SV, international physical activity questionnaire-short version; GPAQ, global physical activity questionnaire; ACE I/D, Angiotensin-Converting enzyme Insertion/Deletion; BFM, body fat mass; BMI, body mass index; LPA, light physical activity; MVPA, moderate-to-vigorous physical activity; WC, waist circumference; WHR, waist-to-hip ratio; FM%, fat mass percentage; ASMM, appendicular skeletal muscle mass

**Table 2:** Quality assessment results of results included studies using the JBI critical appraisal checklist

Cross-sectional studies (Author, year)	Q1	Q2	Q3	Q4	Q5	Q6	Q7	Q8	Q9	Q10	Q11	Total (%)
Aggio DA, 2016	Y	Y	Y	Y	Y	Y	Y	Y				100.00%
Batista PP, 2023	Y	Y	U	Y	Y	Y	Y	Y				93.75%
Da Silva JRD, 2018	Y	U	U	Y	Y	Y	Y	Y				87.50%
Escriba-Salvans A 2022	Y	Y	Y	Y	Y	Y	Y	Y				100.00%
Gianoudis J, 2015	Y	Y	U	Y	Y	Y	Y	Y				93.75%
Meier NF, 2020	Y	Y	U	Y	Y	Y	Y	Y				93.75%
Mo YH, 2022	Y	Y	U	Y	Y	Y	Y	Y				93.75%
Reid N, 2018	Y	U	Y	Y	Y	Y	Y	Y				93.75%
Ribeiro SV, 2020	Y	Y	U	Y	Y	Y	Y	Y	Y	U	Y	90.90%
Sánchez JL, 2019	Y	Y	Y	Y	Y	Y	Y	Y				100.00%
Scott D, 2021	Y	Y	Y	Y	Y	Y	Y	Y				100.00%
Senior HE, 2015	Y	Y	U	Y	Y	Y	Y	Y				93.75%
Sinesio Silva L, 2016	Y	Y	Y	Y	Y	Y	Y	Y				100.00%
Smith L, 2020	Y	Y	U	Y	Y	Y	Y	Y				93.75%
Taani MH, 2021	Y	Y	Y	Y	Y	Y	Y	Y				100.00%
Tzeng PL, 2020	U	U	U	Y	Y	Y	Y	Y				81.25%
Yao XM, 2022	Y	Y	U	Y	Y	Y	Y	Y				93.75%

Q: question; Y: yes; U: unclear

**Table 3:** Subgroup analysis of the associations between sarcopenia and sedentary behaviour with different skeletal muscle mass measures, and physical performance measures

The subgroups	Studies, n	*I*^*2*^, %	OR value	Heterogeneity	
				95% CI	*P* value
**SMM measures**					
Equation	3	56.4	1.32	0.77–2.25	0.101
None	2	68.6	2.87	1.47–5.60	0.064
DXA	3	1.0	1.17	1.00-1.35	0.364
BIA	6	76.4	1.34	1.05–1.69	0.006
**PP measures**					
GS	8	71.0	1.81	1.03–1.36	0.001
None	2	68.6	2.87	1.47–5.60	0.074
SPPB	2	0.0	1.18	0.98–1.40	0.916
TUG	2	0.0	1.33	1.06–1.68	0.900

OR, odd ratio; CI, confidential interval; SMM, skeletal muscle mass; DXA, dual-energy X-ray absorptiometry; BIA, bioelectrical impedance analysis; PP, physical performance; GS, gait speed; SPPB, short physical performance battery; TUG, time up to go.

### Electronic supplementary material

Below is the link to the electronic supplementary material.

**Supplementary Material 1:** PRISMA Reporting Checklist 2020

Supplementary Material 2

Supplementary Material 3

## Data Availability

The data used and analysed during the current study are available from the corresponding author on reasonable request.

## List of abbreviations

ACE I/D: Angiotensin-Converting enzyme Insertion/Deletion
ASMM: Appendicular skeletal muscle mass
AWGS: Asian Working Group for Sarcopenia
BFM: Body fat mass
BIA: Bioelectrical impedance analysis
BMI: Body mass index
CI: Confidence intervals
CPM: Counts per minutes
DXA: dual-energy X-ray absorptiometry
EMA: Ecological momentary assessment
EWGSOP: European Working Group on Sarcopenia in Older Persons
FM%: Fat mass percentage
FNIH: The Foundation for the National Institutes of Health
GS: Gait speed
GPAQ: Global physical activity questionnaire
HGS: Hand grip strength
HR: Hazard Risk
IPAQ: International Physical Activity Questionnaire
IPAQ-SV: International physical activity questionnaire-short version
JBI: Joanna Briggs Institute
IADL: Instrumental Activity of Daily Living
LMM: Low muscle mass
LMS: Low muscle strength
LPP: Low physical performance
LPA: Light physical activity
MeSH: Medical Subject Heading
MVPA: Moderate-to-vigorous physical activity
OR: Odd Ratio
PASE: Physical activity scale for the elderly
PRISMA 2020: Preferred Reporting Items for Systematic Reviews and Meta-Analyses 2020 statement
RR: Relative Risk
SD: Standard deviation
SMM: Skeletal muscle mass
SPPB: Short physical performance battery
SARC-F: The strength, assistance in walking, rising from a chair, climbing stairs, and falls questionnaire
TUG: Time up to go
WC: Waist circumference
WHR: Waist-to-hip ratio
