# Supplementary material for: The association between sedentary behaviour and sarcopenia in older adults: a systematic review and meta-analysis
Source: BMC Geriatr. 2023 Dec 20;23:877. doi: 10.1186/s12877-023-04489-7 (PMC10734096; doi:10.1186/s12877-023-04489-7)
Supplement: Supplementary file 2 — Supplementary Material 2 [file 12877_2023_4489_MOESM2_ESM.docx]

**Number of results for each database**

| **Database** | **Number of results** |
| --- | --- |
| **MEDLINE** | **751** |
| **Embase** | **1225** |
| **PsycInfo** | **1430** |
| **CINAHL** | **505** |
| **Web of Science** | **606** |
| **Wanfang** | **144** |
| **CNKI** | **67** |
| **Sinomed** | **37** |
| **Total** | **4765** |

**08^th^ Aug. 2023**

**Search strategy**

| **Table 1** | **Search strategy in MEDLINE database via Ovid** |
| --- | --- |
| 1 | exp Sarcopenia/ or exp Muscular atrophy/ or exp Muscle weakness/ |
| 2 | (sarcopenia* or sarcopenias* or muscle atrophy* or muscle atrophies* or muscular atrophy* or muscular atrophies* or muscle weakness* or muscular weakness*).ti,ab. |
| 3 | (muscle mass* or muscular mass*).mp. |
| 4 | exp Muscle strength/ or exp Hand strength/ |
| 5 | (muscle strength* or hand strength* or hand strengths* or grip* or grips* or grasp* or grasps*).ti,ab. |
| 6 | exp Physical functional performance/ or exp Walking speed/ |
| 7 | (functional performance* or functional performances* or physical performance* or physical performances* or physical functional performance* or physical functional performances* or gait speed* or gait speeds* or walking pace* or walking paces* or walking speed* or walking speeds*).ti,ab. |
| 8 | 1 or 2 or 3 or 4 or 5 or 6 or 7 |
| 9 | exp Sedentary behavior/ or exp Screen time/ |
| 10 | (sedentary behavior* or sedentary lifestyle* or sedentary time* or sedentary times* or screen time* or screen times*).ti,ab. |
| 11 | (sedentariness* or sitting time* or excessive sitting*).mp. |
| 12 | 9 or 10 or 11 |
| 13 | exp Aged/ or exp Home nursing/ or exp Long-term care/ or exp Independent living/ |
| 14 | (aged* or elderly* or home care* or long term care* or community dwelling* or independent living).ti,ab. |
| 15 | (older adults* or older people* or older persons* or convalescence home* or extended care facility* or long term care facility* or skilled nursing facility* or long term medical care* or long term therapy* or long term treatment* or community dwelling* or community-dwelling* or independent living).mp. |
| 16 | 13 or 14 or 15 |
| 17 | 8 and 12 |
| 18 | 16 and 17 |

| **Table 2** | **Search strategy in Embase database via Ovid** |
| --- | --- |
| 1 | exp Sarcopenia/ or exp Muscular atrophy/ or exp Muscle weakness/ |
| 2 | (sarcopenia* or muscle atrophy* or muscle atrophies* or muscular atrophy* or muscular atrophies* or muscle degeneration* or muscle recession* or muscle wasting* or muscular degeneration* or myoatrophy* or myodegeneration* or myophagism* or muscle strength loss* or muscle weakening* muscular weakness* or neuromuscular fatigue*).ti,ab. |
| 3 | exp Muscle mass/ |
| 4 | (muscle volume*).ti,ab. |
| 5 | (skeletal muscle mass* or skeletal muscle index*).mp. |
| 6 | exp Muscle strength/ or exp Grip strength/ or exp Hand grip/ |
| 7 | (muscle power* or muscular dynamic strength* or muscle dynamic strength* or muscular force* or muscular strength* grasp force* or grasp forces* grasp strength* or grasping strength* or gripping force* or gripping power* or handgrip strength* or handgrip*).ti,ab. |
| 8 | exp physical performance/ or exp walking speed/ or exp gait/ |
| 9 | (physical functional performance* or physical ability* or physical performance* or physical performancy* or gait speed* or gait velocity* or stride speed* or stride velocity* or walk speed* or walk velocity* or walking rate* or walking velocity*).ti,ab. |
| 10 | 1 or 2 or 3 or 4 or 5 or 6 or 7 or 8 or 9 |
| 11 | exp Sedentary lifestyle/ or exp Sedentary time/ or exp Screen time/ |
| 12 | (sedentary behavior* or sedentary behaviour* or sedentary life style* or sitting time* or time spent sitting* or screen viewing time* or screen watching times*).ti,ab. |
| 13 | (sedentariness* or excessive sitting*).mp. |
| 14 | 11 or 12 or 13 |
| 15 | 10 and 14 |
| 16 | exp Aged/ or exp nursing home/ or exp long term care/ or community dwelling person/ |
| 17 | (aged* or elderly* or convalescence home* or extended care facility* or long term care facility* or skilled nursing facility* or long term medical care* or long term therapy* or long term treatment* or community dwelling individuals* or community dwelling participants* or community dwelling people* or community dwelling persons* or community dwelling subjects* or community-dwelling cohort*).ti,ab. |
| 18 | (older adults* or older people* or older persons*).mp. |
| 19 | 16 or 17 or 18 |
| 20 | 15 and 19 |

| **Table 3** | **Search strategy in APA Psyclnfo database via Ovid** |
| --- | --- |
| 1 | exp Muscular atrophy/ or exp Muscular Disorders/ or exp Musculoskeletal Disorders/ |
| 2 | (muscular atrophy* or muscular disorders* or musculoskeletal disorders*).ti,ab. |
| 3 | (sarcopenia* or muscle weakness* or muscular mass* or skeletal muscle index*).mp. |
| 4 | exp Physical strength/ |
| 5 | (physical strength* or hand strength* or hand grip strength* or hand grasp strength*).ti,ab. |
| 6 | exp Physical activity/ or exp Walking/ or exp Gait/ |
| 7 | (physical activity* or walking speed* or gait speed* or walking pace*).ti,ab. |
| 8 | (physical function* or physical functional performance* or physical performance* or walking speed* or gait speed* or walking pace*).mp. |
| 9 | 1 or 2 or 3 or 4 or 5 or 6 or 7 or 8 |
| 10 | exp Sedentary behavior/ or exp Screen time/ |
| 11 | (sedentary behavior* or screen time*).ti,ab. |
| 12 | (sedentary lifestyle* or sedentary time* or sedentariness* or sitting time* or excessive sitting*).mp. |
| 13 | 10 or 11 or 12 |
| 14 | 9 and 13 |
| 15 | exp Aged/ or exp Long term care/ or exp Nursing homes/ or Communities/ |
| 16 | (aged* or long term care* or nursing home* or communities).ti,ab. |
| 17 | (older adults* or older people* or older persons* elderly* or long-term care* or nursing home care* or convalescence home* or extended care facility* or long term care facility* or skilled nursing facility* or long term medical care* or long term therapy* or long term treatment* or community dwelling individuals* or community dwelling participants* or community dwelling people* or community dwelling persons* or community dwelling subjects* or community-dwelling cohort*).mp. |
| 18 | 15 or 16 or 17 |
| 19 | 14 and 18 |

| **Table 4** | **Search strategy in CINAHL database via EBSOhost** |
| --- | --- |
| S1 | (MH “Sarcopenia”) OR (MH “Muscular atrophy”) OR (MH “Muscle weakness”) |
| S2 | TI ( sarcopenia* or sarcopenias* or muscle atrophy* or muscle atrophies* or muscular atrophy* or muscular atrophies* or muscle weakness* or muscular weakness* ) OR AB ( sarcopenia* or sarcopenias* or muscle atrophy* or muscle atrophies* or muscular atrophy* or muscular atrophies* or muscle weakness* or muscular weakness* ) |
| S3 | (MH "Muscle Strength") OR (MH "Grip Strength") |
| S4 | TI ( muscle strength* or hand strength* or grip strength* or grasp strength* ) OR AB ( muscle strength* or hand strength* or grip strength* or grasp strength* ) |
| S5 | (MH "Physical Performance") OR (MH "Walking Speed") |
| S6 | TI ( functional performance* or physical performance* or physical functional performance* or gait speed* or walking pace* or walking speed* ) OR AB ( functional performance* or physical performance* or physical functional performance* or gait speed* or walking pace* or walking speed* ) |
| S7 | S1 OR S2 OR S3 OR S4 OR S5 OR S6 |
| S8 | (MH "Life Style, Sedentary") OR (MH "Screen Time") |
| S9 | TI ( sedentary behavior* or sedentary lifestyle* or sedentary time* or screen time* ) OR AB ( sedentary behavior* or sedentary lifestyle* or sedentary time* or screen time* ) |
| S10 | S8 OR S9 |
| S11 | (MH "Aged") OR (MH "Health Services for the Aged") OR (MH "Rehabilitation, Geriatric") OR (MH "Gerontologic Nursing") OR (MH "Gerontologic Care") OR (MH "Community Health Centers") |
| S12 | TI ( aged* or elderly* or older adults* or older people* or older persons* or community dwelling individuals* or community dwelling participants* or community dwelling people* or community dwelling persons* or community dwelling subjects* or community-dwelling cohort*) OR AB ( aged* or elderly* or older adults* or older people* or older persons* or community dwelling individuals* or community dwelling participants* or community dwelling people* or community dwelling persons* or community dwelling subjects* or community-dwelling cohort*) |
| S13 | S12 OR S13 |
| S14 | S7 AND S10 AND S13 |

| **Table 5** | **Search strategy in Web of Science** |
| --- | --- |
| Web of Science Core Collection | (TS=(Sarcopenia) AND TS=(Sedentary behavior or Sedentary lifestyle or Sedentary time or Sitting time) AND TS=(Aged or Older adults or Older people or Older persons)) |

| **Table 6** | **Search strategy in CNKI (China National Knowledge Infrastructure)** |
| --- | --- |
| Professional search | (SU=(sarcopenia + sarcopenic + muscle wasting) OR SU=(muscle mass + skeletal muscle mass + skeletal muscle index) OR SU=(muscle strength + hand grip strength) OR SU=(physical performance+ gait speed + six-minute walking test)) AND (SU=sedentary lifestyle + sedentary behaviour + sedentary time + prolonged sitting) AND (SU=elderly + older adults) |

| **Table 7** | **Search strategy in WanFang** |
| --- | --- |
| Professional search (subject word expansion) | (Title or keyword: (sarcopenia or sarcopenic or muscle wasting) or Subject: (muscle mass or skeletal muscle mass or skeletal muscle index) or Subject: (muscle strength or hand grip strength) or Subject: (Physical performance or gait speed or six-minute walking test)) and (Title or keyword: (sedentary lifestyle or sedentary time or sedentary behaviour or sedentary time or prolonged sitting)) |

| **Table 8** | **Search strategy in Sinomed** |
| --- | --- |
| **Advanced search** | |
| #1 | ("sarcopenia" OR "Sarcopenic" OR "muscle wasting" OR ("muscle mass" OR "skeletal muscle mass" OR "skeletal muscle index" OR "muscle strength" OR "hand grip strength" OR "physical performance" OR "gait speed" OR "six-minute walking test" |
| #2 | ("sedentary lifestyle" OR "sedentary behaviour" OR "sedentary time" OR "prolonged sitting") |
| #3 | #1 AND #2 |
