# Supplementary material for: The association between sedentary behaviour and sarcopenia in older adults: a systematic review and meta-analysis
Source: BMC Geriatr. 2023 Dec 20;23:877. doi: 10.1186/s12877-023-04489-7 (PMC10734096; doi:10.1186/s12877-023-04489-7)
Supplement: Supplementary file 3 — Supplementary Material 3 [file 12877_2023_4489_MOESM3_ESM.docx]

**
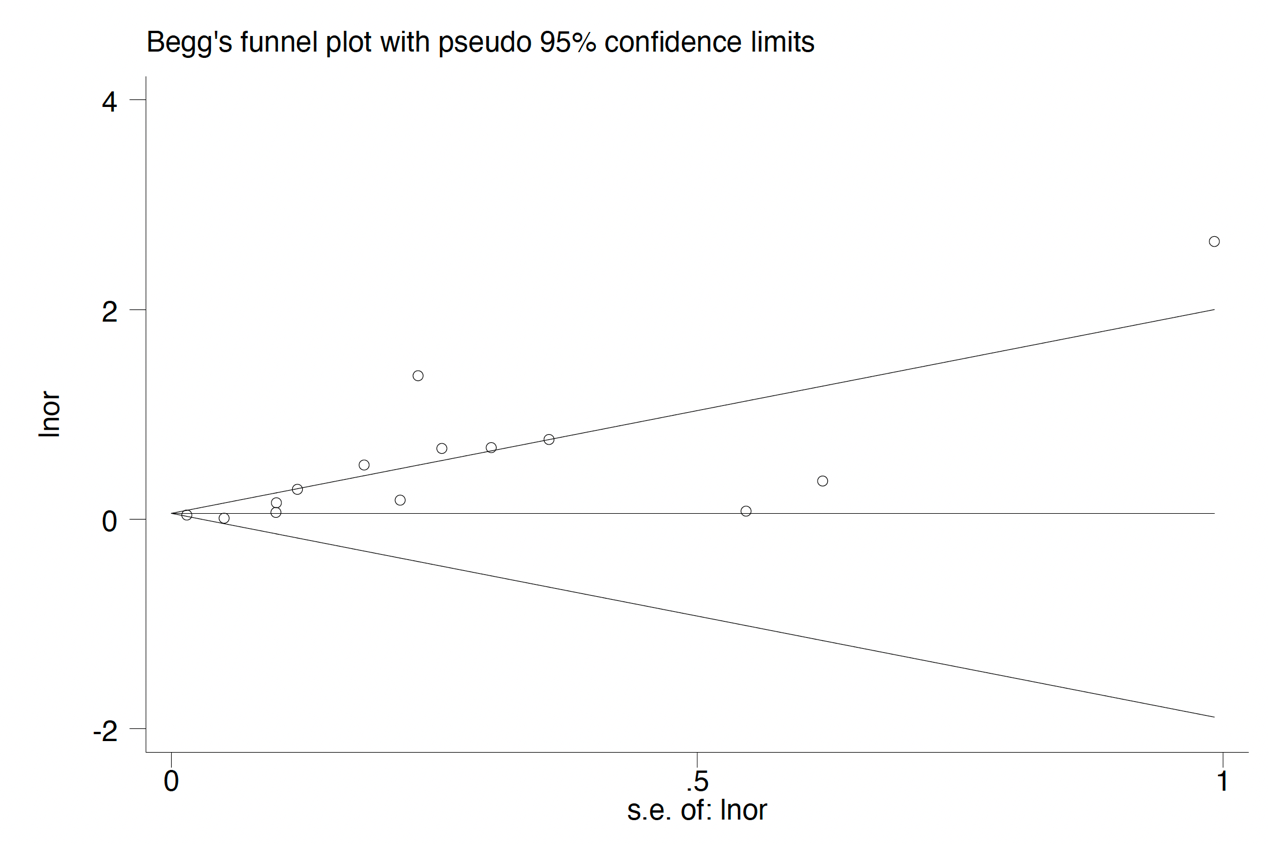
**

**Supplementary material 3.** Begg’s funnel plot for the association between sedentary behaviour and sarcopenia
